# Role of β-Lactamase Inhibitors as Potentiators in Antimicrobial Chemotherapy Targeting Gram-Negative Bacteria

**DOI:** 10.3390/antibiotics13030260

**Published:** 2024-03-15

**Authors:** Song Zhang, Xinyu Liao, Tian Ding, Juhee Ahn

**Affiliations:** 1 Department of Biomedical Science, Kangwon National University, Chuncheon 24341, Republic of Korea; ed5988449@kangwon.ac.kr; 2 Future Food Laboratory, Innovation Center of Yangtze River Delta, Zhejiang University, Jiaxing 314100, China; xinyu_liao@zju.edu.cn; 3 Department of Food Science and Nutrition, Zhejiang University, Hangzhou 310058, China

**Keywords:** antibiotic resistance, β-lactamase inhibitor, β-lactam, combination therapy

## Abstract

Since the discovery of penicillin, β-lactam antibiotics have commonly been used to treat bacterial infections. Unfortunately, at the same time, pathogens can develop resistance to β-lactam antibiotics such as penicillins, cephalosporins, monobactams, and carbapenems by producing β-lactamases. Therefore, a combination of β-lactam antibiotics with β-lactamase inhibitors has been a promising approach to controlling β-lactam-resistant bacteria. The discovery of novel β-lactamase inhibitors (BLIs) is essential for effectively treating antibiotic-resistant bacterial infections. Therefore, this review discusses the development of innovative inhibitors meant to enhance the activity of β-lactam antibiotics. Specifically, this review describes the classification and characteristics of different classes of β-lactamases and the synergistic mechanisms of β-lactams and BLIs. In addition, we introduce potential sources of compounds for use as novel BLIs. This provides insights into overcoming current challenges in β-lactamase-producing bacteria and designing effective treatment options in combination with BLIs.

## 1. Introduction

In recent decades, the evolution and rapid spread of antibiotic-resistant bacteria have threatened public health and become a major threat worldwide. In the U.S. alone, antibiotic-resistant infections have been estimated to cause more than 23,000 deaths and USD 35 billion in economic losses every year [1]. Although the development of antibiotic agents has been ongoing for several decades, the clinical supply of novel antibiotics remains unsatisfactory due to the rapid emergence of antibiotic-resistant bacteria [2]. The overuse and misuse of antibiotics in clinics and animal farms have exerted excessive selective pressure on the bacterial ecosystem, leading to the rapid dissemination of antibiotic resistance [3]. Moreover, limited chemotherapeutic options lead to frequent treatment failure in cases of pan-drug-resistant bacterial infections. Therefore, the discovery of novel antibiotics in the clinical pipeline is an essential strategy to effectively control antibiotic-resistant bacteria [4]. β-lactam antibiotics (BLAs) are most commonly used to treat bacterial infections, accounting for nearly 60% of global antibiotic usage, due to their broad spectrum of antimicrobial activity [5,6]. BLAs comprise four major categories, including penicillin, cephalosporins, monobactams, and carbapenems. These antibiotics have a common structural feature, the four-membered β-lactam ring. *N*-acetylmuramic acid (NAM) units linked to pentapeptides are major cell wall components [7]. Cross-linking *D*-alanine-*D*-alanine NAM pentapeptides catalyzed by penicillin-binding proteins (PBPs) can confer rigidity and osmotic stability to the bacterial cell wall [8]. The β-lactam ring exhibits structural similarity to *D*-Ala-*D*-Ala peptides [9], which can irreversibly acylate the serine-active sites of PBPs and block further transpeptidation reactions, resulting in a damaged cell wall and bacterial death [10].

**Figure 1:**
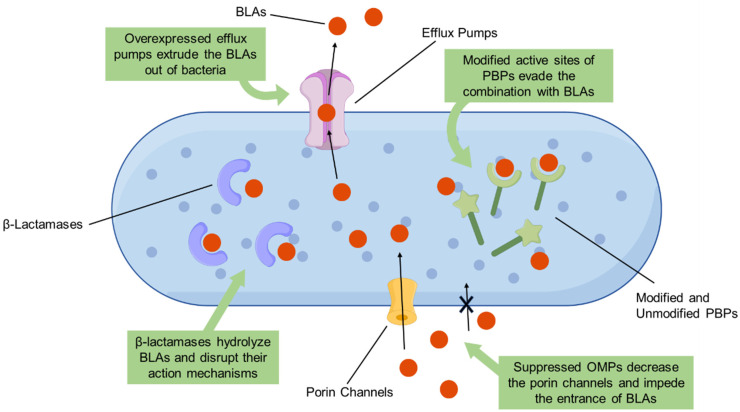
Proposed bacterial mechanisms of resistance to β-lactam antibiotics (BLAs), including the production of β-lactamases, the modified active sites of penicillin-binding proteins (PBPs), and down-regulated outer membrane proteins (OMPs).

Unfortunately, the evolution of antibiotic resistance in pathogens has significantly reduced the effectiveness of BLAs. The World Health Organization (WHO) has designated a priority list of antibiotic-resistant bacteria, including *Acinetobacter baumannii*, *Pseudomonas aeruginosa*, and *Enterobacteriaceae* [11] (Table 1). The resistance mechanisms of bacteria to BLAs are the production of β-lactamases, the modified active sites of PBPs, the down-regulation of outer-membrane proteins (OMPs), and the overexpression of efflux pumps [7] (Figure 1). Among these mechanisms, the production of β-lactamases is a major cause of the resistance of Gram-negative bacteria to BLAs [12]. Bacteria-produced β-lactamases can hydrolyze BLAs that bind to PBPs, preserving the physiological function and structure of the cell wall [13,14]. Furthermore, the mutation of PBPs is the most representative resistance mechanism in Gram-positive bacteria [12]. The modified active sites of PBPs can effectively reduce affinity for BLAs, resulting in enhanced resistance. For example, the altered forms of PBPs can induce BLA resistance in bacteria and transfer to susceptible bacteria to develop antibiotic resistance [15]. The down-regulation of OMPs, such as OprD in *P*. *aeruginosa* and CarO in *A*. *baumannii*, can reduce membrane permeability and prevent the binding of BLAs to PBPs [16,17]. Notably, the down-regulation of OMPs is not sufficient to enhance bacterial resistance to BLAs [7]. Efflux pumps contribute to multidrug resistance by transporting antibiotics outside the cell, which can synergistically increase the carbapenem resistance mediated by β-lactamases [18].

Recently, the rapid emergence and widespread distribution of BLA resistance have been mainly attributed to β-lactamase-producing Gram-negative bacteria, resulting in multidrug and pan-drug resistance [19]. In order to deal with this threat, two main strategies have been proposed to restore the antimicrobial activity of BLAs against bacteria: (i) the development of novel BLAs that can resist the hydrolysis of β-lactamase and (ii) the discovery of β-lactamase inhibitors (BLIs) that can be used as adjuvants to improve the antibiotic efficacy of BLAs [7]. However, the development of new BLAs and the assessment of their clinical feasibility are time-consuming and costly processes. Furthermore, bacteria can easily evolve resistance to newly developed BLAs. However, BLIs are not antibiotics, which may not cause antibiotic resistance in bacteria. BLIs target the active sites of β-lactamases and inhibit the formation of complexes between BLAs and β-lactamases. Hence, the antibiotic activity of BLAs can be restored when combined with BLIs, which can increase the possibility of re-using BLAs [20]. For instance, BLA–BLI combinations such as amoxicillin–clavulanic acid, ampicillin–sulbactam, and piperacillin–tazobactam have been extensively utilized and can significantly enhance antibiotic activity. This review discusses the classification and characteristics of different classes of BLAs, the synergistic inhibitory mechanisms of BLAs and BLIs, and the potential sources of compounds for use as novel BLIs.

**Table 1:** The priority list of antibiotic-resistant bacteria declared by the WHO.

Priority	Gram-Staining Category	Bacteria	Major BLA Resistance	Reference
Priority 1: Critical	Negative	*Acinetobacter baumannii*	Carbapenem	[21]
	Negative	*Pseudomonas aeruginosa*	Carbapenem	[22]
	Negative	*Enterobacteriaceae*	Carbapenem, 3rd Generation of Cephalosporin	[23,24]
Priority 2: High	Positive	*Enterococcus faecium*	Vancomycin	[25]
	Positive	*Staphylococcus aureus*	Methicillin, Vancomycin	[26]
	Negative	*Helicobacter pylori*	Clarithromycin	[27]
	Negative	*Campylobacter* spp.	Fluoroquinolone	[28]
	Negative	*Salmonellae* spp.	Fluoroquinolone	[29]
	Negative	*Neisseria gonorrhoeae*	Cephalosporin, Fluoroquinolone	[30]
Priority 3: Medium	Positive	*Streptococcus pneumoniae*	Penicillin	[2]
	Negative	*Haemophilus influenzae*	Ampicillin	[31]
	Negative	*Shigella* spp.	Fluoroquinolone	[32]

WHO: World Health Organization; BLA: β-lactam antibiotics.

## 2. Classification of β-Lactamases and Genetic Transfer Origins

Previous studies have shown that β-lactamases can hydrolyze the β-lactam ring through acylation/deacylation and thereby deactivate penicillin, cephalosporins, monobactams, and carbapenems [20]. According to the β-lactamase database, more than 8100 β-lactamases have been identified and classified based on their structural and functional properties; the Ambler molecular classification uses amino acid sequences, and the Bush–Jacoby–Medeiros functional classification uses biochemical substrates and inhibitory profiles [6,10,33] (Table 2). Specifically, the Ambler molecular classification categorizes serine-β-lactamases (SBLs) into classes A, C, and D, while metallo-β-lactamases (MBLs) are classified into class B [34]. The chromosome- and plasmid-encoded β-lactamase genes can be transferable, causing the emergence of β-lactamase-producing bacteria [7,19].

The class A enzymes, according to the Ambler classification, are predominant β-lactamases, showing a broad spectrum of activity. There are two common plasmid-mediated β-lactamases, designated TEM-1 and SHV-1, found in most *Escherichia coli*, *Neisseria gonorrhoeae*, *H. influenzae*, and *P. aeruginosa* [6]. These enzymes are capable of inactivating penicillin and narrow-spectrum cephalosporins. Up to now, new antibiotics such as penems and cephems have been used to treat β-lactam-resistant bacteria [7]. Nevertheless, bacteria produce extended-spectrum β-lactamases (ESBLs) under antibiotic selective pressure, resulting in the inactivation of extended-spectrum cephalosporins and monobactam aztreonam [42]. Among ESBLs, more than 246 have been identified as belonging to the CTX-M family, which is involved in the regulation of chromosomes, plasmids, and mobile genetic elements [33,35]. The CTX-M family shows greater enzymatic activity than other ESBLs against third-generation cephalosporins such as cefotaxime, ceftriaxone, and ceftazidime [43]. Interestingly, these ESBLs do not degrade carbapenems, and their enzymatic activity is inhibited by common BLIs such as clavulanic acid, sulbactam, and tazobactam.

The class A carbapenemases that hydrolyze carbapenems belong to the KPC, GES, NMC-A, IMI, and SME families [44]. The KPC and GES enzymes are the common plasmid-borne carbapenemases, widely distributed in *Klebsiella pneumoniae*, *A. baumannii*, *Salmonella* spp., and *Enterobacter* spp. [45]. KPC-type β-lactamases are highly resistant to clavulanic acid, sulbactam, and tazobactam, while these are suppressed by avibactam and vaborbactam [46]. Unlike KPC-type β-lactamases, NMC-A, IMI, and SME enzymes are chromosome-encoded carbapenemases, predominantly identified in *Enterobacter cloacae* and *Serratia marcescens* [10]. These carbapenemases are responsible for the transfer of resistance genes through plasmid mobilization [37].

Ambler class C AmpC β-lactamases are the predominate cephalosporinases encoded on both chromosomes and plasmids [40]. AmpC β-lactamases mainly include the CMY, FOX, MIR, ACT, and DHA families, which are widely distributed in multiple Gram-negative bacteria such as *Citrobacter freundii*, *Morganella morganii*, *P. aeruginosa*, and *E. cloacae* [23]. AmpC β-lactamase-producing bacteria are able to hydrolyze penicillin, cephalosporins (except cefepime), aztreonam, and cephamycin [47]. The majority of AmpC β-lactamases have no carbapenemase activity. However, mutated AmpC β-lactamases down-regulate OMPs and up-regulate efflux pumps, leading to resistance to carbapenem antibiotics [43,48]. AmpC β-lactamases are resistant to β-lactam-based inhibitors but sensitive to diazabicyclo-octane BLIs and boronate BLIs [49].

Ambler class D enzymes, also known as oxacillinases, are characterized by their ability to hydrolyze isoxazolylpenicillins. Commonly, plasmid-encoded OXA β-lactamases are resistant to clavulanic acid, sulbactam, and tazobactam, conferring resistance to penicillin, cephalosporins, and carbapenems [41]. OXA β-lactamases, including OXA-23, OXA-40, OXA-48, OXA-58, and OXA-143, can degrade carbapenems and are produced by in *A. baumannii*, *P. aeruginosa*, and *Enterobacteriaceae* [50,51]. Moreover, most OXA β-lactamases are known as carbapenemases because of their ability to increase carbapenem resistance in low-permeability environments, especially in *A. baumannii* [52].

In contrast to SBLs, which possess catalytic serine residues, Ambler class B enzymes, as MBLs, use zinc ions to mediate antibiotic hydrolysis. The hydrolytic spectrum of MBLs encompasses most BLAs, with the exception of aztreonam [53]. Although MBLs are resistant to most available BLIs, their hydrolyzing activity is easily inhibited by chelating agents [10]. According to their amino acid sequences and active site characteristics, MBLs can be further divided into three different subclasses: B1, B2, and B3. Among these, subclass B1 is included in plasmid-borne MBLs such as the IMP, VIM, and NDM families, which are widely distributed in *A. baumannii*, *P. aeruginosa*, and *K. pneumoniae* [38]. Notably, NDM, a unique membrane-bound protein, can protect neighboring bacteria from antibiotics and transfer resistance genes through outer-membrane vesicles [54]. In contrast, the subclasses B2 and B3 encompass some chromosome-encoded MBLs, such as CphA in *Aeromonas hydrophila*, L1 in *Stenotrophomonas maltophilia*, and GOB-1, in *Chryseobacterium meningosepticum* [39].

## 3. Criteria for Selecting Appropriate BLIs as a Promising Antimicrobial Strategy

BLIs have gained great attention due to their effectiveness in inhibiting β-lactamases. The application of BLIs can be a promising strategy to control antibiotic-resistant bacteria in clinical settings, specifically for β-lactamase-producing bacteria. Recently, BLIs combined with β-lactam antibiotics were used to improve antibiotic activity against β-lactamase-producing bacteria. A few BLIs have been approved for clinical use. For example, a ceftolozane–tazobactam combination was used to clinically treat complicated intra-abdominal infections caused by ESBL-producing *Enterobacteriaceae* and *P. aeruginosa* [55]. The combination of ceftazidime–avibactam showed a synergistic effect on the treatment of complicated urinary tract infections and intra-abdominal infections caused by ceftazidime-resistant *Enterobacteriaceae* [56]. The development of BLIs is still in the experimental stages and undergoing clinical trials. Moreover, the currently approved combinations are encountering resistance issues, such as clinical strains developing multiple drug resistance mechanisms, including alterations in amino acid sequences, decreases in membrane permeability, mutations in target protein mutations, and the activation in efflux pumps [57]. Therefore, the discovery of BLIs is essential to increasing the utilization of current antibiotics and designing new treatment options for antibiotic-resistant bacterial infections in clinical practice.

**Figure 2:**
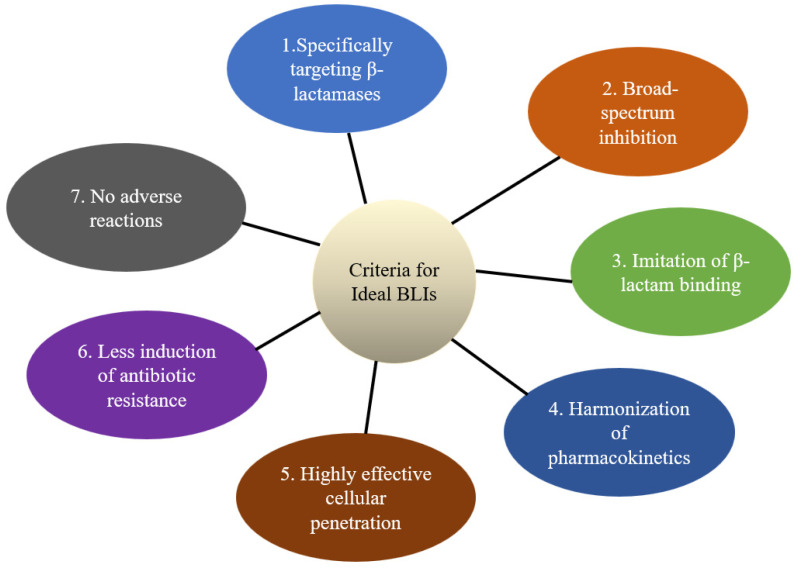
Criteria for ideal β-lactamase inhibitors (BLIs).

Many potential compounds for BLIs have been evaluated and verified to enhance the activity of β-lactam antibiotics in laboratories and clinical trials. The criteria for selecting suitable BLIs include (1) the specificity of BLIs for β-lactamases, (2) the broad-spectrum inhibition of BLIs against β-lactamases, (3) the synergy of BLIs with β-lactams, (4) the optimum pharmacokinetic properties of BLIs, (5) the high-efficiency cellular penetration of BLIs, (6) less induction of BLI resistance, and (7) no adverse effect of BLIs (Figure 2). BLIs need to specifically and competitively target β-lactamases by mimicking β-lactam antibiotics. For example, avibactam effectively inhibits ESBL activity in multidrug-resistant *Enterobacteriaceae* and *P. aeruginosa* [58]. Similarly, tazobactam is a non-toxic inhibitor responsible for class A β-lactamases [59]. Hence, the specific binding to β-lactamases plays a key role in restoring the efficacy of β-lactam antibiotics. Currently, SBLs and MBLs can degrade many β-lactam antibiotics. Therefore, it is necessary to develop broad-spectrum BLIs that inhibit SBLs and MBLs. In general, β-lactamases show high affinity for substrates such as penicillin analogs and cephalosporin analogs [60]. This implies that BLIs with structures similar to these antibiotics can competitively inhibit β-lactamase activity. The optimal pharmacokinetic properties of BLIs ensure adequate concentrations at the infection site, which can restore antibiotic activity [61,62]. Mutations in porins can result in antibiotic resistance in Gram-negative bacteria [63]. For instance, the overexpression of *bla*_KPC_ can mediate outer-membrane permeability, leading to avibactam resistance [64,65]. Thus, the ability of BLIs to penetrate outer membranes is also a critical criterion because β-lactamases are located in the bacterial periplasmic space. Non-toxicity is one of the most important criteria for employing BLIs in clinical practice. In this context, novel BLIs need to be evaluated for off-target effects causing side effects. 

## 4. Application of BLIs and Non-BLIs

### 4.1. Classical Penicillin-Based BLIs

As early BLIs, clavulanic acid, sulbactam, and tazobactam have been successively developed to restore antibiotic activity against bacteria. Specifically, these BLIs can competitively bind to β-lactamases to enhance the antibiotic activity of β-lactams. These BLIs involve the irreversible binding of serine residues at the β-lactamase active site, resulting in enzyme inactivation [38]. The inhibitory spectrum of early BLIs primarily targets Ambler class A β-lactamases such as TEM-1, TEM-2, SHV-1, and CTX-M but shows no inhibitory effect against SBLs and MBLs due to the alteration of active sites [66,67]. Recently, clavulanic acid, sulbactam, and tazobactam have been used in combination with antibiotics in clinical trials (Table 3).

Clavulanic acid is a β-lactam compound derived from *Streptomyces clavuligerus* and was the first BLI in clinical practice [68]. The combination of amoxicillin and clavulanic acid has been used in treatments of bacterial infections in the gut, bacteremia after dental procedures, chronic bronchitis, and acute otitis media. Clavulanic acid and amoxicillin have a half-life of approximately 1 h and exhibit similar distribution patterns [69]. However, the use of clavulanic acid in oral infections can lead to an increase in gastrointestinal side effects. Hence, the combination ratio of amoxicillin to clavulanic acid is typically 4:1 to minimize the risk of potential toxicity and to ensure inhibitory activity against β-lactamase-producing bacteria [70]. Additionally, drug-induced hepatitis can cause a potential side effect in combination therapy because the liver metabolizes clavulanic acid [71].

Penicillin-based BLIs such as sulbactam were synthesized after the discovery of clavulanic acid. Ampicillin–sulbactam is a commonly used combination in clinical practice for the treatment of intra-abdominal and skin infections and the prevention of postoperative infections. Commonly, the half-life of ampicillin and sulbactam is roughly 1 h with renal metabolism [72]. Unlike clavulanic acid, sulbactam is typically administered parenterally due to its poor absorption efficiency when administrated orally [7]. Sulbactam has intrinsic antibacterial activity against *Acinetobacter* and *Bacteroides*. However, it is not effective against *A*. *baumannii* mutated in penicillin-binding protein 3 (PBP3) [73]. Furthermore, sulbactam shows a lower inhibitory effect on the enzymatic activity of TEM-1 and SHV-1 than clavulanate and tazobactam [74].

Tazobactam is also a synthetic penicillin sulfone introduced to the market as a third BLI [75]. Piperacillin–tazobactam is one of the most effective combinations and shows a broad spectrum of antibacterial activity. In general, the optimum ratio of piperacillin to tazobactam is 8:1, with an elimination half-life of 0.8–1 h [76]. This combination therapy has been widely applied against bloodstream infections, febrile neutropenia, diabetic foot infections, and septic shock. The common side effects include headache, nausea, diarrhea, and hypersensitivity reactions [77]. However, piperacillin–tazobactam typically is not effective against non-class A enzyme-producing *A*. *baumannii* and *S*. *maltophilia* [78]. In recent years, a new combination of ceftolozane and tazobactam was developed to combat multidrug-resistant infections. Its broad spectrum of antibacterial activity can used as an alternative to the use of carbapenem antibiotics [79]. According to a recent report, however, bacteria can evolve resistance to the ceftolozane–tazobactam combination [80].

Enmetazobactam, also known as AAI101, is a N-methylated derivative of tazobactam. Unlike tazobactam, enmetazobactam has an extra methyl group at the triazole ring that improves antibiotic activity [81]. As a new penicillanic acid sulfone, enmetazobactam exhibits potent inhibitory activity against SBLs, particularly class C and D enzymes in *Enterobacteriaceae* [82,83]. In contrast, no inhibitory activity has been observed in *S*. *maltophilia*, *A*. *baumannii*, or AmpC-producing *P*. *aeruginosa* [84]. Cefepime–enmetazobactam is the most common combination used in clinical practice. Enmetazobactam can restore bacterial susceptibility to cefepime from 2% to 98%, especially against class A enzyme-producing bacteria [85]. Currently, the cefepime–enmetazobactam combination is applied for phase 2 and 3 clinical trials to treat UTIs.

### 4.2. First Generation of Non-BLIs: Diazabicyclooctanes

Despite the effective inhibition of early BLIs against class A β-lactamases, these BLIs still lack sufficient potency to control infections caused by MBLs, AmpC-type enzymes, and carbapenemases. Recently, diazabicyclooctanes (DBOs) were developed to treat BLI-resistant bacterial infections. DBOs are non-β-lactam compounds that reversibly acylate the active site of serine residue against β-lactamase [86]. DBOs can deactivate class A, C, and D enzymes. Nonetheless, MBLs still evade inhibition owing to their distinctive active sites [63]. Relevant clinical trials are undergoing to explore the potential of DBOs in combination with other antibiotics (Table 3).

Avibactam, also known as AVE1330A or NXL104, is the first SBL inhibitor belonging to the diazabicyclooctanes. Avibactam is mostly effective against class A and C β-lactamases of *Enterobacteriaceae* and *P*. *aeruginosa* [66,87]. Ceftazidime–avibactam is one of the most prevalent combinations, having been evaluated in phase 3 and phase 4 trials for the treatment of cystic fibrosis, hospital-acquired bacterial pneumonia (HABP), urinary tract infections (UTIs), and acute pyelonephritis. Avibactam is mainly cleared via the kidney with a 1.7–2.1 h half-life [88]. Moreover, relatively few adverse effects have been observed with the administration of avibactam, except for rare headaches, nausea, and diarrhea [89]. Avibactam alone is not effective against *A*. *baumannii* and MBL-producing bacteria, whereas the combination of avibactam and aztreonam can inhibit SBLs and MBLs [90].

Relebactam, formerly designated as MK-7655, shares a structural similarity with avibactam, characterized by the addition of a piperidine ring [91]. Relebactam is effective against class A and C β-lactamases and less effective against class D enzymes [92]. The combination of imipenem–cilastatin–relebactam is currently undergoing two phase 4 clinical trials for the treatment of cystic fibrosis and bacterial pneumonia. This combination therapy can further enhance efficacy and safety compared with colistin monotherapy [93].

Nacubactam, also called RG6080 or OP05095, has a carbamoyl side chain that includes an additional aminoethoxy group [94]. Currently, the efficacy of the meropenem–nacubactam combination is being evaluated in two phase 3 clinical trials for the treatment of complicated urinary tract infections (cUTIs), acute pyelonephritis, HABP, ventilator-associated bacterial pneumonia (VABP), and complicated intra-abdominal infections (cIAIs). In general, nacubactam is well tolerated and occasionally leads to headaches or complications associated with intravenous access [43].

Zidebactam, previously known as WCK 5107, is a DBO inhibitor with PBP2 binding activity [10]. The cefepime–zidebactam combination shows strong inhibitory effects against β-lactamases produced by *P*. *aeruginosa*, *A*. *baumannii*, and *Enterobacteriaceae* [95]. Currently, the cefepime–zidebactam combination is undergoing phase 3 clinical trials to evaluate its efficacy in treating cUTIs and acute pyelonephritis.

Durlobactam, also named ETX2514, inhibits most β-lactamases, except for class B [96]. Unlike conventional combinations, durlobactam is commonly used in combination with sulbactam. This therapy has been evaluated in four clinical trials for the treatment of cUTIs, acute pyelonephritis, and *A*. *baumannii* infections. Durlobactam can penetrate outer-membrane protein A (OmpA). Thus, the down-regulation of OmpA can confer resistance to durlobactam in pathogens [97].

Funobactam, formerly designated as XNW4107, is a novel DBO inhibitor developed in recent years. Funobactam is effective against class A, C, and D enzymes, particularly the OXA-24/40-like β-lactamases produced by *A*. *baumannii* [98]. Notably, funobactam has no intrinsic antibacterial activity and is commonly used in combination with imipenem [99]. Recently, the combination of imipenem and funobactam was evaluated in two phase 3 clinical trials for the treatment of HABP, VABP, and cUTIs.

### 4.3. Second Generation of Non-BLIs: Boronic Acid Derivatives

Phenylboronic acid was first used to deactivate penicillinases, and then, boronic acids were widely investigated as inhibitors for combatting distinct β-lactamases. These BLIs are designed as transition state analogs that can reversibly bind to active sites and competitively inhibit β-lactamases [38]. Unlike conventional BLIs and diazabicyclooctanes, boronic acids can suppress SBLs and MBLs. In addition, there is still no information that describes the hydrolytic activity of β-lactamases on boronic acids [100]. Currently, clinical trials have been conducted to identify the potency of combination therapy utilizing boronic acids and antibiotics (Table 3).

Vaborbactam, formerly known as RPX7009, is known as the first boronic-acid-related BLI targeting KPC-type carbapenemases and other class A and C β-lactamases [101]. Vaborbactam primarily enters bacteria through OmpK35 and OmpK36, responsible for binding and acylating the active sites of enzymes [102]. In general, vaborbactam by itself has no antibacterial activity, but the meropenem–vaborbactam combination is used for therapeutic purposes in clinical practice. Currently, phase 3 clinical trials are being conducted to evaluate the efficacy of this combination in treating cUTIs, acute pyelonephritis, and HABP. Additionally, the therapeutic benefits of this combination include high clinical cure rates, low mortality rates, and reduced nephrotoxicity [103]. However, the meropenem–vaborbactam combination is not effective in the treatment of infections caused by *A*. *baumannii* and *P*. *aeruginosa*, which may involve alternative resistance mechanisms such as porin alterations and efflux activity [104].

Taniborbactam, also named VNRX-5133, can inhibit SBL and MBL activities, and is known as the first pan-spectrum BLI capable of inhibiting class A, B, C, and D β-lactamases [105]. Commonly, taniborbactam has a half-life ranging from 3.5 to 4.8 h, metabolized in the kidney [106]. Recently, two clinical trials (phase 1 and phase 3) were completed using a cefepime–taniborbactam combination for the treatment of UTIs and acute pyelonephritis. Taniborbactam has great potential as an inhibitor when combined with proper antibiotics [107]. However, the application of taniborbactam can cause side effects consisting of dizziness, headache, nausea, and diarrhea [108].

Xeruborbactam, also known as QPX7728, can inhibit a variety of MBLs and SBLs, including KPC-, AmpC-, and OXA-type enzymes [109,110]. In contrast to vaborbactam and taniborbactam, xeruborbactam has antibacterial activity [111]. As a pan-spectrum BLI, xeruborbactam in combination with antibiotics can suppress β-lactamases and effectively control bacterial infections. For instance, the meropenem–xeruborbactam combination is synergistically effective against multiple β-lactamase-producing *K*. *pneumoniae* [112]. Recently, two phase 1 clinical trials were conducted to evaluate the pharmacokinetics and side effects of xeruborbactam in bacterial infections. Additionally, QPX7728 can be administered orally in combination with other oral antibiotics in clinical practice [113].

In general, the utilization of BLIs in clinical settings has been gradually recognized as having great prospects in β-lactamase inhibition. Different generations of BLIs can demonstrate synergistic effects in combination with BLAs, including enhancing inhibitory activity, extending inhibitory ranges, and maintaining BLA levels in the body. Unfortunately, adverse reactions due to BLIs have been witnessed in clinical trials. These inhibitors can result in certain nervous system dysfunctions, gastrointestinal reactions, and anaphylactic reactions, such as clavulanic acid, avibactam, tazobactam, nacubactam, and taniborbactam. Specifically, the β-lactam ring can target side chains and cause cross-reactivity with some BLAs, leading to anaphylactic reactions [114]. Similarly, mild headaches, confusion, and seizure risk have also been reported in some cases involving high doses or poor metabolism [115]. The hepatorenal toxicity induced by BLIs also requires more attention. The elimination route of BLIs is closely related to hydrophilia, and thus, hydrophilic BLIs are almost metabolized by the kidney. Notably, the combination of hydrophilic BLIs and BLAs can increase metabolic burden, including ceftaroline–avibactam, imipenem–relebactam, and cefepime–zidebactam [116,117,118]. To avoid metabolic toxicity, it is imperative to consider the specific doses used for patients with liver and kidney dysfunction.

## 5. Inhibitory Enzymatic Mechanisms of SBLs and MBLs

SBLs and MBLs have distinct enzymatic mechanisms that can inactivate antibiotics due to their structural and functional characteristics. SBLs hydrolyze β-lactam antibiotics via catalytic sites containing serine residue. The interaction of β-lactams and SBLs forms hydrogen bonds through a process of acylation and deacylation, resulting in the release of inactive antibiotics and active β-lactamases [119]. MBLs produce nucleophiles at the active sites in the presence of Zn^2+^ and amino acids and attack the β-lactam ring, resulting in the hydrolysis of β-lactam antibiotics [10]. Therefore, BLIs need various inhibitory mechanisms to target the distinct hydrolytic activities of SBLs and MBLs, which can restore the efficacy of β-lactam antibiotics. Specifically, SBL inhibitors commonly target and bind to the serine residue at the catalytic site to protect β-lactam antibiotics from hydrolysis, while MBL inhibitors can competitively bind to active sites or chelate zinc ions to inactivate MBLs.

**Figure 3:**
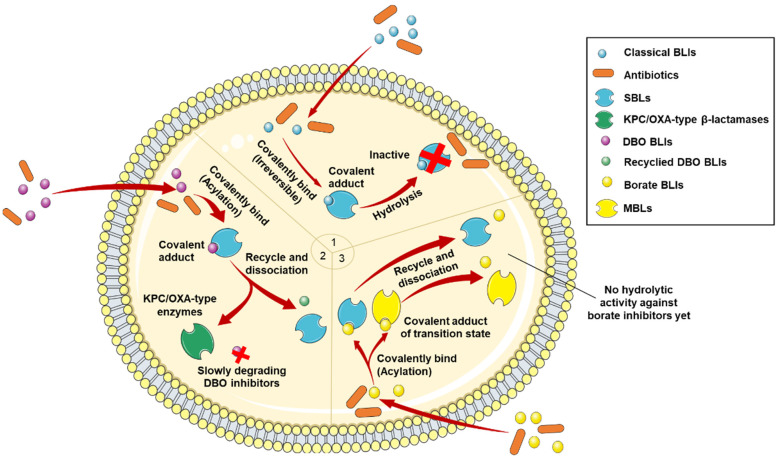
Main inhibitory mechanisms of β-lactamase inhibitors (BLIs). 1: The classical BLIs are mainly characterized as suicide inactivators, combining with serine residue at active sites and inactivating β-lactamases. 2: The first generation of non-β-lactam diazabicyclooctane (DBO) BLIs mediate reversible acylation reactions to inhibit SBLs. 3: The second generation of non-β-lactam borate BLIs can form a covalent adduct transition state to reversibly inhibit both serine-β-lactamases (SBLs) and metallo-β-lactamases (MBLs). KPC and OXA denote *Klebsiella pneumoniae* carbapenemase and oxacillin-hydrolyzing enzymes, respectively.

Current research has proposed three types of BLIs characterized by distinct inhibitory mechanisms (Figure 3). The early BLIs function as suicide inactivators by forming a covalent adduct with serine residue and effectively binding to SBLs [38]. Subsequently, the BLI molecule undergoes gradual hydrolysis and fragmentation, resulting in the inactivation of the β-lactamase adduct [120]. However, these mechanisms cannot effectively inhibit ESBLs and suppress carbapenemases and MBLs. Thus, DBO inhibitors have been developed to treat BLI-resistant bacterial infections. Unlike suicide inactivators, DBO inhibitors reversibly bind to β-lactamases, enabling re-cyclization and resulting in sustained inhibitory effects [86,121]. Unfortunately, bacterial resistance to DBO inhibitors can be developed due to the slow inactivation process of DBO inhibitors against β-lactamases [122].

A broader spectrum of novel BLIs can be developed with structural modifications. For instance, the incorporation of a piperidine ring into the carbamoyl side chain can generate a positive charge in relebactam under physiological pH, leading to the reduced extrusion of BLIs from bacterial cells and enhanced antibacterial activity against β-lactamase-producing bacteria [43,91]. Likewise, the addition of an aminoethoxy group into the carbamoyl side chain can induce the intrinsic antibiotic activity of nacubactam [84,94]. Boronic acid can form an enzyme–inhibitor complex, competitively inhibiting SBLs and MBLs [107,111]. Specifically, boronic acid BLIs reversibly form coordinate covalent bonds with SBLs to induce a transition state to mimic the β-lactamase hydrolytic reaction. Hence, the primary mechanism of action for boronic acid BLIs is the mimicry of tetrahedral intermediates during the enzymatic hydrolysis of SBLs and MBLs, leading to the restoration of antibiotic efficacy.

Various chelating agents, such as aspergillomarasmine A, thiol-based compounds, and phosphonate-containing compounds, can be potential BLIs against MBLs [123,124,125]. These agents directly chelate zinc ions at active sites. Moreover, BLI ANT431 can penetrate the bacterial periplasm where MBLs are produced and inhibit enzyme activity [107,126]. However, BLI ANT431 is limited in use due to its toxicity, off-target effects, differential pharmacokinetics, and drug interactions.

In addition to the enzymatic inhibitory activity of BLIs, intrinsic bacteriostatic activity has also been widely investigated in association with PBPs [111]. For example, avibactam can directly suppress *Enterobacter* strains by inhibiting PBP2 activity [127]. Similarly, sulbactam can inhibit PBP1 and PBP3 in *A*. *baumannii*, resulting in the inactivation of bacteria [128]. Therefore, the combination of intrinsic bacteriostatic activity and enzymatic inhibitory activity may contribute to better antibacterial potency and extend the inhibitory spectrum. However, the potential intrinsic bacteriostatic activity of BLIs may induce the up-regulation of antibiotic resistance genes, leading to an increase in β-lactamase activity.

## 6. Potential Sources of BLIs

### 6.1. Novel Synthetic BLIs

Phosphonates are categorized as organophosphorus compounds that have abundant biological activities, including antibacterial, antiviral, anti-cancer, and anti-inflammatory activities [129]. Phosphonate derivatives can inhibit class A, C, and D enzymes via acylation reactions at serine active sites [130,131]. Phosphonate derivates have shown the specific and time-dependent suppression of class C β-lactamases in *Enterobacter cloacae* P99 [132]. Similarly, in preclinical studies, MG96077, a novel phosphonate BLI, was combined with imipenem, showing a significant reduction of 90% in MIC against imipenem-resistant *P*. *aeruginosa* and *K*. *pneumoniae* [100]. Recently, a metal-binding pharmacophore was discovered, conferring inhibitory activity from phosphonates against MBLs such as IMP-1, NDM-1, and VIM-5 [133]. However, phosphonate derivates are unstable in aqueous solutions and susceptible to phosphodiesterase, resulting in limited clinical prospects.

Monobactams are antibiotic agents produced by pathogens [134]. The representative product is the synthetic compound aztreonam, which is used to treat bacterial infections in clinical settings. In addition, aztreonam can also suppress class C β-lactamases [135]. MK-8712, a monobactam, shows great potency in enhancing imipenem activity against *P*. *aeruginosa* [136]. A siderophore monobactam, Syn2190, combined with ceftazidime or cefpirome can suppress AmpC-producing *Enterobacteriaceae* and *P*. *aeruginosa* [137]. Nonetheless, Syn2190 exhibits low affinity toward class A enzymes and poses risks in inducing the overexpression of *bla*_AmpC_ [7,100]. BAL30072 is the other type of siderophore and possesses inhibitory activity against antibiotic-resistant bacteria capable of producing carbapenemases or MBLs [138,139]. Additionally, BAL30072 shows intrinsic antibacterial activity and shares bacterial iron transport systems [140].

Dicarboxylate derivatives chelate metal ions, indicating potential MBL inhibitors. Specifically, the carboxylate groups of these compounds chelate Zn^2+^ [141]. Dicarboxylate derivatives combined with imipenem can effectively inhibit MBL-producing bacteria that express NDM, IMP, and VIM [20]. ANT431 is a newly developed pyridine-2-carboxylic acid that suppresses MBLs. ANT431 can penetrate bacterial periplasms where MBLs are produced [126]. ANT431 can restore meropenem activity against carbapenem-resistant *Enterobacteriaceae* [142]. However, the limited-spectrum inhibition of this inhibitor may induce off-target effects by blocking zinc-containing mammalian enzymes [143].

### 6.2. Natural BLIs

Plant extracts are good sources for discovering potential BLIs. For instance, methyl cinnamate derived from *Ocimum basilicum* exhibits similar inhibitory activity against enzymes to clavulanic acid. Specifically, methyl cinnamate targets the same active-site Arg254 as clavulanic acid, and they show similar IC_50_ values when inhibiting the CTX-M β-lactamase [144]. As a natural compound with low cytotoxicity, carnosic acid has been widely utilized in different areas, such as medicine, food, and cosmetics [145]. Studies have shown that carnosic acid can effectively bind to the allosteric sites of NDM-1, such as Phe46, Tyr64, Leu65, Asp66, and Thr94. Ultimately, the bioactivity of NDM-1 will be attenuated, leading to the restored antimicrobial effect of meropenem [146]. Furthermore, phenolic acids such as salicylsalicylic acid, SB-202742, and EGCg are widely distributed in plants, possess various biological activities, and can effectively suppress β-lactamases in a dose-dependent manner [147,148,149]. Notably, natural polyphenols such as proanthocyanidins have been demonstrated to not only suppress SBLs and MBLs but can also overcome PBP2a-mediated β-lactam resistance [150]. In view of the abundant sources of natural compounds and their good properties, they have the potential to become good candidates for BLIs.

Naturally occurring fungal products can be used as BLIs against β-lactamase-producing bacteria such as *Enterobacteriaceae*, *Acinetobacter*, and *Pseudomonas* strains. Specifically, aspergillomarasmine A is nontoxic and inhibits MBLs and carbapenemases in a dose-dependent manner [151]. Currently, most existing BLIs in combination therapy tend to realize SBL inhibition. In contrast, fungus-derived aspergillomarasmine A provides a unique approach to tackling multiple MBL-mediated infections and other carbapenem-resistant pathogens. Therefore, aspergillomarasmine A can be a promising BLI candidate and exert synergistic activity when combined with carbapenem. Although natural extracts are potential sources of BLIs, these compounds—for instance, 1,4-naphthalenedione—may induce cytotoxicity, sore throat, abdominal pain, and vomiting [152]. Given the diversity and complexity of natural extracts, more comprehensive and in-depth experiments are needed to confirm their cytotoxicity and their interactions with BLAs before clinical practice.

## 7. Concluding Remarks and Future Prospects

In conclusion, β-lactamases such as ESBLs, carbapenemases, AmpC, and MBLs pose a significant threat to human health due to their contribution to antibiotic resistance. Although antibiotics have alleviated the pressure associated with bacterial infections, the emergence and spread of antibiotic-resistant bacteria have led to the development of novel antibiotics. However, bacteria quickly evolve resistance to novel antibiotics. Thus, antibiotics alone are insufficient in combating antibiotic resistance. In this context, the use of existing antibiotics combined with BLIs shows significant promise in combating β-lactamase-producing bacteria. Recently, many BLIs have received approval for clinical use because BLIs have broad-spectrum inhibition, high affinity, no toxicity, and excellent pharmacokinetic properties. BLIs and antibiotics are currently available only as fixed-dose combinations in clinical settings. Nevertheless, the emergence of bacterial resistance to BLIs has continuously increased when combined with antibiotics. Hence, it is essential to carefully use current combination therapies and simultaneously explore novel strategies against β-lactamase-producing bacteria. Recently, triplet combination therapy with a BLI and two antibiotics has been proposed to treat BLI-resistant bacterial infections. New scaffolds for BLIs have been developed based on a core structure mainly consisting of β-lactam, DBO, and boronic acid, which includes phenolic acid, monobactam, and phosphonates. Combination therapy involving BLIs shows promise in extending the effectiveness of current antibiotics and improving their efficacy in tackling the challenges of the post-antibiotic era. Due to the complexity and interactions of BLI candidates, it is necessary to further investigate their mechanisms, pharmacokinetics, and cytotoxicity.

## Figures and Tables

**Table 2:** Classification schemes and characteristics of β-lactamases.

Ambler Classification	Functional Scheme	Representative Enzyme	Relevant Bacteria	Transfer Origin	Substrate	Reference
Class A	2b	TEM-1, TEM-2, and SHV-1	*E*. *coli*, *N*. *gonorrhoeae*, *P*. *aeruginosa*, *H*. *influenzae*, and *K*. *pneumoniae*	Plasmid-mediated	Penicillin and narrow-spectrum cephalosporins	[6]
	2be	CTX-M, SHV-2, TEM-10, and GES-1	*E*. *coli*, *K*. *pneumoniae*, *C*. *freundii*, and other *Enterobacteriaceae*	Chromosome-encoded and plasmid-mediated	Penicillin, aztreonam, narrow and extended-spectrum cephalosporins	[33,35]
	2br	TEM-30, SHV-72	*K*. *pneumoniae*, *E*. *coli*,	Plasmid-mediated	Penicillin	[7]
	2c	PSE (CARB)	*V*. *cholerae*, *E*. *coli*, *S*. *enterica*, *P*. *aeruginosa*, and *S*. *typhimurium*	Chromosome-encoded	Penicillin and carbenicillin	[36]
	2f	KPC-2, KPC-3, GES-2, GES-11, NMC-A, IMI-1, SFC-1, and SME-1	*K*. *pneumoniae*, *E*. *cloacae*, *A*. *baumannii*, *P*. *aeruginosa*, and *S*. *marcescens*	Chromosome-encoded and plasmid-mediated	Penicillin, cephalosporins, aztreonam, and carbapenems	[10,37]
Class B	3	IMP, VIM, NDM, CphA, and L1	*A*. *baumannii*, *P*. *aeruginosa*, *K*. *pneumoniae*, *A*. *hydrophila*, and *S*. *maltophilia*	B1: Plasmid-mediated (mainly) B2 and B3: chromosome-encoded	Penicillin, cephalosporins, and carbapenems	[38,39]
Class C	1	CMY, FOX, MIR, ACT, and DHA	*C*. *freundii*, *E*. *cloacae*, *S*. *marcescens*, *P*. *aeruginosa*, *K*. *pneumoniae*, and *M*. *morganii*	Chromosome-encoded and plasmid-mediated	Penicillin, aztreonam, cephamycin, and cephalosporins (except cefepime)	[40]
Class D	2d	OXA-23, OXA-40, OXA-48, OXA-51, and OXA-58	*A*. *baumannii*, *E*. *coli*, *E*. *cloacae*, *K*. *pneumoniae*, *P*. *aeruginosa*, and other *Enterobacteriaceae*	Plasmid-mediated (mainly) and chromosome-encoded	Penicillin, cephalosporins, and carbapenems	[10,41]

*A*. *baumannii*, *Acinetobacter baumannii*; *A*. *hydrophila*, *Aeromonas hydrophila*; *C*. *freundii*, *Clostridium freundii*; *E*. *coli*, *Escherichia coli*; *E*. *cloacae*, *Enterobacter cloacae*; *H*. *influenzae*, *Haemophilus influenzae*; *K*. *pneumoniae*, *Klebsiella pneumoniae*; *M*. *morganii*, *Morganella morganii*; *N*. *gonorrhoeae*, *Neisseria gonorrhoeae*; *P*. *aeruginosa*, *Pseudomonas aeruginosa*; *S*. *marcescens*, *Serratia marcescens*; *S*. *maltophilia*, *Stenotrophomonas maltophilia*; *S*. *enterica*, *Salmonella enterica*; *S*. *typhimurium*, *Salmonella typhimurium*; *V*. *cholerae*, *Vibrio cholerae*.

**Table 3:** Clinical application of combination therapy: BLIs and antibiotics.

β-Lactamase Inhibitor	Chemical Structure	Company	Clinical Trial Phase	Representative Combination	Indication
Clavulanic acid	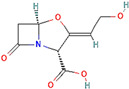	GlaxoSmithKline (London, the UK)	Phase 4	Amoxicillin–Clavulanic Acid	Bacteremia (NCT02783404) Chronic Bronchitis (NCT00656747) Effects on Gut Microbiota (NCT04084106) Acute Otitis Media (NCT00644943)
Sulbactam	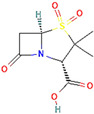	Pfizer Inc. (New York, NY, USA)	Phase 4	Ampicillin–Sulbactam	Cesarean Section (NCT01138852) Intra-Abdominal Infection (NCT00952796) Acinetobacter Pneumonia (NCT05922124) Skin Infections (NCT00368537)
Tazobactam	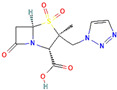	Taiho Pharmaceutical Co., Ltd. (Tokyo, Japan)	Phase 4	Piperacillin–Tazobactam Ceftolozane–Tazobactam	Bloodstream Infections (NCT05355350) Febrile Neutropenia (NCT04233996) Diabetic Foot Infections (NCT00044746) Early Phase of Severe Sepsis and Septic Shock (NCT02730624) Cystic Fibrosis and Bronchiectasis (NCT06035055)
Enmetazobactam	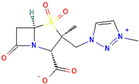	Allecra Therapeutics (Saint Louis, MO, USA)	Phase 3 Phase 2	Cefepime–Enmetazobactam	Urinary Tract Infection Complicated (NCT03687255, NCT05826990, NCT03680612)
Avibactam	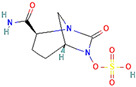	Pfizer Inc. (New York, NY, USA)	Phase 4	Ceftazidime–Avibactam	Cystic Fibrosis (NCT02504827) Hospital-Acquired Pneumonia (NCT04774094) Urinary Tract Infection and Acute Pyelonephritis (NCT04882085)
Relebactam	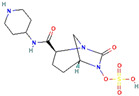	Merck Sharp & Dohme LLC (Rahway, NJ, USA)	Phase 4	Imipenem–Cilastatin–Relebactam	Cystic Fibrosis and Bacterial Pneumonia (NCT05561764) Obesity and Critical Illness (NCT05146154)
Nacubactam	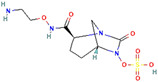	F. Hoffmann-La Roche, Ltd. (Basel, Switzerland)	Phase 1	Meropenem–Nacubactam	Gram-Negative Bacterial Infections (NCT03182504)
Zidebactam	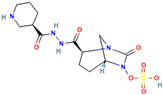	Medpace, Inc. (Cincinnati, OH, USA)	Phase 3	Cefepime–Zidebactam	Complicated Urinary Tract Infections and Acute Pyelonephritis (NCT04979806)
Durlobactam	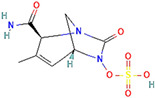	Entasis Therapeutics Holdings Inc. (Waltham, MA, USA)	Phase 3 Phase 2	Sulbactam–Durlobactam	Hospital-Acquired Bacterial Pneumonia (NCT03894046) Complicated Urinary Tract Infections and Acute Pyelonephritis (NCT03445195)
Funobactam	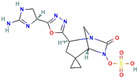	Evopoint Biosciences Inc. (Suzhou, China)	Phase 3	Imipenem–Funobactam	Complicated Urinary Tract Infection Including Acute Pyelonephritis (NCT05204368) Hospital-Acquired Bacterial Pneumonia or Ventilator-Associated Bacterial Pneumonia (NCT05204563)
Vaborbactam	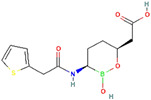	Melinta Therapeutics, Inc. (Parsippany, NJ, USA)	Phase 3	Meropenem–Vaborbactam	Complicated Urinary Tract Infection and Acute Pyelonephritis (NCT02166476) Hospital-Acquired Bacterial Pneumonia (NCT02168946)
Taniborbactam	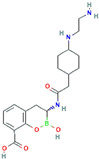	Venatorx Pharmaceuticals, Inc. (Malvern, PA, USA)	Phase 3 Phase 1	Cefepime–Taniborbactam	Complicated Urinary Tract Infection and Acute Pyelonephritis (NCT03840148) Pharmacokinetics (NCT04951505)
Xeruborbactam	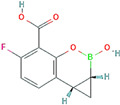	Qpex Biopharma, Inc. (San Diego, CA, USA)	Phase 1	QPX2014–Xeruborbactam Ceftibuten–Xeruborbactam	Pharmacokinetics and Side Effects (NCT05072444, NCT04380207) Bacterial Infections (NCT06079775)

## Data Availability

Not applicable.
